# Swing-Leg Trajectory of Running Guinea Fowl Suggests Task-Level Priority of Force Regulation Rather than Disturbance Rejection

**DOI:** 10.1371/journal.pone.0100399

**Published:** 2014-06-30

**Authors:** Yvonne Blum, Hamid R. Vejdani, Aleksandra V. Birn-Jeffery, Christian M. Hubicki, Jonathan W. Hurst, Monica A. Daley

**Affiliations:** 1 Department of Comparative Biomedical Sciences, Royal Veterinary College, Hatfield, Hertfordshire, United Kingdom; 2 Mechanical, Industrial and Manufacturing Engineering, Oregon State University, Corvallis, Oregon, United States of America; 3 Department of Biology, University of California Riverside, Riverside, California, United States of America; Delft University of Technology (TUDelft), Netherlands

## Abstract

To achieve robust and stable legged locomotion in uneven terrain, animals must effectively coordinate limb swing and stance phases, which involve distinct yet coupled dynamics. Recent theoretical studies have highlighted the critical influence of swing-leg trajectory on stability, disturbance rejection, leg loading and economy of walking and running. Yet, simulations suggest that not all these factors can be simultaneously optimized. A potential trade-off arises between the optimal swing-leg trajectory for disturbance rejection (to maintain steady gait) versus regulation of leg loading (for injury avoidance and economy). Here we investigate how running guinea fowl manage this potential trade-off by comparing experimental data to predictions of hypothesis-based simulations of running over a terrain drop perturbation. We use a simple model to predict swing-leg trajectory and running dynamics. In simulations, we generate optimized swing-leg trajectories based upon specific hypotheses for task-level control priorities. We optimized swing trajectories to achieve i) constant peak force, ii) constant axial impulse, or iii) perfect disturbance rejection (steady gait) in the stance following a terrain drop. We compare simulation predictions to experimental data on guinea fowl running over a visible step down. Swing and stance dynamics of running guinea fowl closely match simulations optimized to regulate leg loading (priorities i and ii), and do not match the simulations optimized for disturbance rejection (priority iii). The simulations reinforce previous findings that swing-leg trajectory targeting disturbance rejection demands large increases in stance leg force following a terrain drop. Guinea fowl negotiate a downward step using unsteady dynamics with forward acceleration, and recover to steady gait in subsequent steps. Our results suggest that guinea fowl use swing-leg trajectory consistent with priority for load regulation, and not for steadiness of gait. Swing-leg trajectory optimized for load regulation may facilitate economy and injury avoidance in uneven terrain.

## Introduction

Legged locomotion involves coordination of limb swing and stance phases with distinct yet tightly coupled dynamics. Studies of legged locomotion often focus primarily on the dynamics of the stance phase, during which an animal's legs experience the greatest demands for force and power [1]–[8]. Yet, recent research highlights the critical role of swing-leg trajectory on locomotor dynamics—experimental evidence shows that leg loading is critically sensitive to the initial landing conditions (leg angle, leg length and body velocity) at the swing-stance transition [9]–[11], which are influenced by swing-leg trajectory. Running animals must effectively coordinate the interplay of swing-leg trajectory, landing conditions and stance leg loading [12]–[16]. For example, when running guinea fowl encounter an unexpected pothole, late-swing leg retraction leads to variation in leg contact angle, which explains 80% of the variance in stance leg impulse [17]. Thus, the swing-leg trajectory is a critical factor in the dynamics of legged locomotion, particularly during movement over uneven terrain.

Recent theoretical studies have highlighted inherent trade-offs in swing-leg trajectory for walking and running in uneven terrain. Simple walking and running models have revealed that swing-leg velocity just before the stance transition influences numerous aspects of locomotor dynamics, including stability [14]–[16], [18], robustness [19], leg work [19], [20], disturbance rejection and collision impact energy losses [18]. Previous studies suggest these factors cannot be simultaneously optimized—resulting in a trade-off between two families of performance objectives: swing-leg velocity can be optimized to minimize peak forces, work and collision impacts [16], [18]–[20], or to provide stability, disturbance rejection and robustness of body centre of mass (CoM) dynamics [15], [16], [18]–[20], but not all simultaneously. Thus, a potential trade-off has emerged between optimal swing-leg trajectory to regulate leg loading for *injury avoidance*, or alternatively, to facilitate steady gait through *disturbance rejection*. Yet, while theoretical studies suggest such a trade-off, there is no experimental data on how running animals optimize swing-leg trajectory for non-steady locomotion.

Do running animals favor one end of this trade-off, or alternatively, find a compromise solution? Both disturbance rejection and injury avoidance have potential to be important task-level priorities for running animals. Disturbance rejection refers to minimizing the effect of perturbations on the body center of mass (CoM) trajectory [21]. Buffering the CoM motion against disturbances reduces the risk of fall, and may minimize need for active control intervention [22], [23]. Furthermore, some experimental evidence has suggested steady CoM dynamics as an important task-level goal in legged locomotion [9], [24]. However, minimizing leg impacts and peak forces may also be critical, because animal legs have relatively constant safety factors in musculoskeletal structures around 2–4× the peak forces of steady locomotion [25], [26]. Perfect disturbance rejection can demand large leg forces [18]–[20], which could lead to musculoskeletal injury. Building legs to withstand very large forces would require carrying extra weight, so limited safety factors in animal legs may reflect a compromise between safety and economy. Specialized runners like cursorial ground birds appear to have a structure that is more optimized for economy, with relatively light legs and thin tendons, which could inherently limit safety factors [6], [26], [27], making them prone to injury [28], [29]. Based on these considerations, we reason that both disturbance rejection and injury avoidance have potential to be important, yet sometimes conflicting, task-level priorities in animal locomotion.

In this paper, we test the hypothesis that running guinea fowl use swing-leg trajectory optimized to regulate leg loading (reflecting priority for injury avoidance), against an alternative hypothesis that they use swing-leg trajectory optimized for disturbance rejection (reflecting priority for steady body dynamics). These hypotheses represent the two ends of the theoretical trade-off in swing-leg trajectory described above, providing useful points of comparison to animal behavior. In reality, animal swing-leg trajectory could reflect an intermediate compromise solution, which can be revealed by comparing experimentally observed swing trajectories to simulation predictions for the two hypothetical extremes. We experimentally measured swing-leg trajectory and stance dynamics of guinea fowl running over a visible step down in terrain, when given ample practice, distance and time to anticipate the drop. This contrasts with previous studies of the intrinsic-dynamic response to an *unexpected* terrain drop [10], [17]. Here, we are focused on understanding the task-level priorities reflected in the ‘optimized’ locomotor behavior.

To generate simulation predictions, we use a simple approach with swing-leg geometry that evolves as a function of time during the flight phase, according to a prescribed trajectory optimized to meet a specific performance objective [20], [30]. The swing-leg trajectory determines the landing conditions at the swing-stance transition, and the landing conditions are used to predict stance dynamics based on a simple running model (see methods for model details). We generate simulations with swing-leg trajectory optimized for three specific performance objectives, the first two reflecting a priority to regulate leg loading, and the third reflecting a priority for disturbance rejection. Specifically, we optimize swing trajectory to achieve i) constant peak force, ii) constant axial impulse, or iii) perfect disturbance rejection (steady gait) in the step immediately following a downward step in terrain. Similar swing-leg control policies have been investigated previously in simulation: Ernst and colleagues investigated swing-leg trajectory optimized to target steady gait (constant speed and bounce height), to provide disturbance rejection in uneven terrain [30], and Vejdani and colleagues compared several possible priorities in simulation, including steady gait, constant leg work and constant leg loading [20]. Here we directly compare simulation predictions to new experimental data on guinea fowl running over a visible step down, to understand how task-level priorities influence swing-leg control in running birds.

Optimization of swing-leg trajectory to achieve well-defined intrinsic-dynamic characteristics could be particularly important for animal locomotion because neuromuscular delays limit the rate of feedback-mediated responses to perturbations, and terrain conditions are not often perfectly known. Neuromuscular delays (synaptic, conduction, electromechanical and force development) can represent a large fraction of the step cycle in animals [10], [31], and therefore limit the rate of feedback in both stance and swing. These neural delays are likely to be especially problematic at the swing-stance transition, when small changes in landing conditions have large influence on stance leg loading and body dynamics [9]–[11]. If the animal's knowledge of the terrain is imperfect, variation in terrain height leads to a disturbance, with the immediate response determined by feed-forward muscle activation and the system's intrinsic dynamics [9], [10], [17]. Application of a prescribed swing-leg trajectory can provide well-defined intrinsic-dynamic response in terms of stability, disturbance rejection and leg loading characteristics, bridging neuromuscular delays and minimizing need for rapid neural feedback. The focus of this paper is to understand the task-level mechanical priorities reflected in the swing-leg trajectory used by running animals.

## Methods

### 1 Experiments

**Figure 1:**
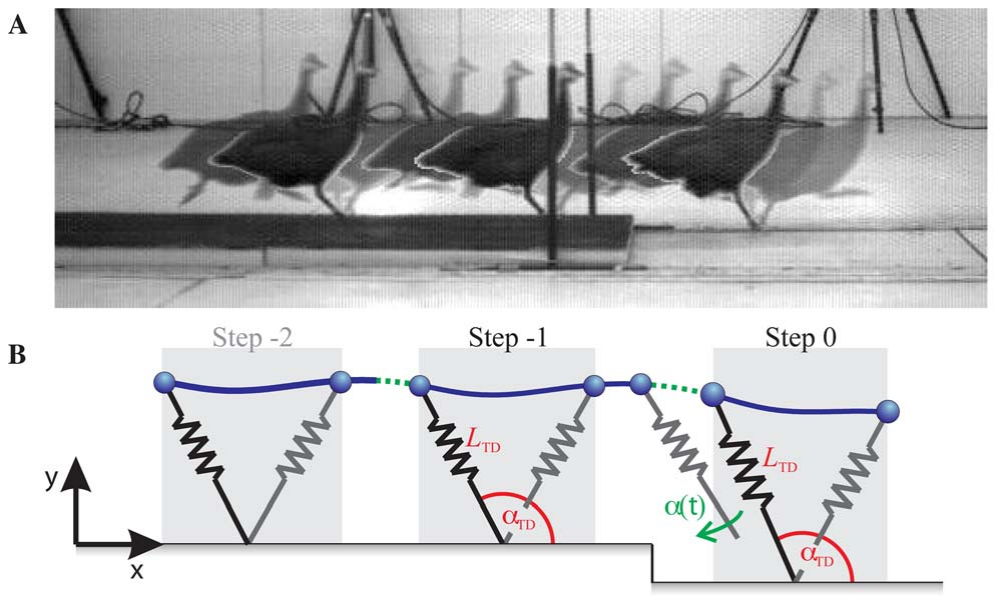
Illustration of experiment and modeling approach. A guinea fowl running a step down (A), and schematic drawing of the spring-loaded inverted pendulum (SLIP) model with swing-leg trajectory control applied as a function of fall time (B). The gray areas indicate the stance phases, and the line represents the body centre of mass (CoM) trajectory. The green dotted line indicates the time between apex and touch down (TD) during which the leg angle of the SLIP is adjusted according to the applied control strategy (see Methods).

Avian running trials were conducted on a 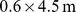 runway. Five 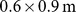 force plates (model 9287B, Kistler, Winterthur, Switzerland) were arranged in a row to record the ground reaction forces (sampling frequency 500 Hz). A camera system (Qualisys, Gothenburg, Sweden), consisting of eight high speed infrared cameras, was used to capture body kinematics (sampling frequency of 250 Hz). For further analysis, both force data and kinematic data were interpolated to a frequency of 500 Hz. We used three experimental terrain conditions: a level runway, a runway with a 4 cm drop and a runway with a 6 cm drop (figure 1(A)).

Five guinea fowl (*Numida meleagris*) (body mass 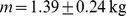, touch down (TD) leg length during level running 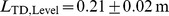) were encouraged to run from one end of the runway to the other (running the step down). We wanted to understand the birds' optimized strategy, as opposed to an unexpected perturbation response, so we trained the birds for a week before data collection. Before data collection, the birds were accustomed both to the task and to being handled by humans. Trials for each terrain condition (level, 4 cm drop, 6 cm drop) were collected in a single block (not randomized), to allow the birds to correctly anticipate the terrain. We collected 10 steady running trials per bird per condition, in which the approach up to the ‘−2 step’ (before the middle of the runway) was in straight-line and approximately steady. Since we could not control the birds’ running speed, we also analyzed their velocity and acceleration during post-processing, as explained in further detail in 2. Neither surgery or anesthesia were used in this study because no invasive procedures were involved. The Royal Veterinary College Ethics and Welfare Committee approved all of the animal experiment protocols under the project title ‘Kinematics and kinetics in birds running over an uneven terrain’.

To approximate the CoM position and the foot point, two markers were attached to the birds' back (cranial and caudal), one at digit III and one at the tarsometatarsophalangeal joint. The marker placement and techniques used to estimate the initial position and velocity of the CoM were the same as reported in [13]. The initial position of the CoM was determined by the average of the cranial and caudal marker position, and the initial velocity condition was derived from kinematics using the path-match optimization technique as described by [17]. We further corrected the initial position estimate based on the assumption that the birds' pitch angular momentum during level running should be minimized (the body should not pitch forward or backward during steady running). This optimization led to an estimate of the true CoM location as positional offsets from the original markers placed on the birds back (horizontal offset 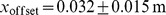, vertical offset 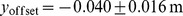) [13]. We then calculated the CoM position trajectories by integrating the ground reaction forces twice.

The following variables were extracted from the experimental data for further analysis: the length of the virtual leg 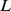, which is defined as the distance between the CoM and the foot point, and its derivative, leg length velocity 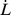 (figure 1(B)), the virtual leg angle 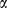, which is measured anti-clockwise with respect to the horizontal, and its derivative, leg angular velocity 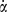, their corresponding TD conditions 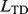 and 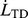, 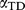, 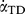, the axial (directed along the virtual leg) and fore-aft horizontal ground reaction force 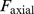 and 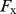, respectively, the axial peak force 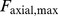, the axial and fore-aft impulse 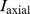 and 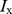, respectively, which are calculated by integrating the corresponding force trajectories over stance time, and the net CoM work 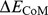, which is the net change in CoM Energy over the course of stance.

### 2 Statistical Analysis

We made all parameters non-dimensional by normalizing them with respect to body mass 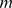, gravitational acceleration 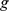, body weight 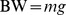, average TD leg length during level running 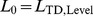 and periodic time of a pendulum 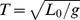. The steps were categorized into four step types: level running (Level), two steps before drop (step −2), the pre-drop step (step −1), the drop step itself (step 0) and the first post-drop step (step +1). Since we could not control the birds' running speed, we analyzed the fore-aft impulse of step −2 during post-processing and selected steady trials (i.e. 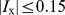 BW 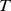, which corresponds to a change in fore-aft velocity of less than 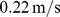) [13]. Step −2 was used only to assess steadiness of the approach, and not further analyzed. We analyzed a total of 367 running steps at speeds between 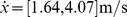 with following sample sizes: Level = 167, Step −1 = 73, Step 0 = 70, and Step +1 = 57.

The statistical analysis of the experimental data was performed in Matlab (R2012a, Mathworks Inc., Natick, MA, USA). We ran a mixed model multi-way ANOVA on the entire dataset with fixed effects ‘step type’ nested within ‘drop height’, ‘individual’ as a random effect and ‘speed’ as a continuous effect (table 1). We then performed post-hoc pair-wise t-tests for the differences between the level mean values and the three step types (−1, 0, and +1), separated into the two drop height conditions (4 cm, 6 cm) (table 2).

**Table 1:** Experimental Data.

		F-ratio		
Parameter		Drop Height(Step Type)	Individual	Speed
*α*_TD_	[deg]	49.1*	140.6*	0.9
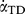	[deg/*T*]	29.8*	36.3*	106.9*
*L*_TD_	[*L*_0_]	15.2*	25.7*	24.6*
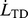	[*L*_0_/*T*]	20.6*	145.3*	259.9*
*k*_Leg_	[BW/*L*_0_]	13.4*	92.2*	8.3*
*F*_axial,max_	[BW]	9.1*	150.3*	67.8*
*I*_axial_	[BW *T*]	8.1*	250.0*	77.1*
*I*_x_	[BW *T*]	15.6*	4.9*	16.5*
Δ*E*_CoM_	[BW *L*_0_]	11.2*	15.9*	19.1*

Analysis of variance (ANOVA) with four factors: Step type nested within drop height, individual as a random effect, and speed as a continuous effect. N = 367 steps. Significant differences (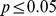) are indicated by asterisks.

**Table 2:** Experimental Data.

Parameter		Level Mean (s.d.)		Drop	Step Type Mean - Level Mean (s.d.)					
					−1		0		+1	
*α*_TD_	[deg]	122.6	(5.4)	4 cm	0.83	(6.0)	−7.1	(6.2)*	−1.8	(6.8)
				6 cm	−0.3	(5.4)	−9.7	(6.0)*	−1.5	(5.6)
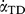	[deg/*T*]	−80.9	(15.5)	4 cm	7.0	(16.6)*	−20.2	(9.6)*	−1.8	(13.1)
				6 cm	2.8	(17.6)	−24.1	(14.2)*	−0.3	(17.8)
*L*_TD_	[*L*_0_]	1.00	(0.03)	4 cm	−0.01	(0.03)*	0.02	(0.03)*	−0.01	(0.04)
				6 cm	−0.02	(0.04)*	0.03	(0.03)*	0.00	(0.04)
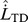	[*L*_0_/*T*]	1.25	(0.22)	4 cm	0.03	(0.22)	−0.18	(0.26)*	0.02	(0.24)
				6 cm	0.01	(0.22)	−0.24	(0.22)*	0.08	(0.26)
*k*_Leg_	[BW/*L*_0_]	11.9	(3.8)	4 cm	3.4	(5.3)*	4.1	(4.2)*	3.3	(4.1)*
				6 cm	3.1	(4.7)*	4.3	(3.9)*	4.3	(4.0)*
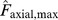	[BW]	2.21	(0.36)	4 cm	0.14	(0.47)	0.10	(0.44)	0.21	(0.49)*
				6 cm	0.04	(0.45)	−0.03	(0.47)	0.33	(0.51)*
*I*_axial_	[BW *T*]	1.01	(0.20)	4 cm	0.04	(0.19)	−0.04	(0.21)	0.02	(0.22)
				6 cm	0.00	(0.26)	−0.14	(0.25)*	0.04	(0.28)
*I*_x_	[BW *T*]	0.01	(0.08)	4 cm	−0.03	(0.07)*	0.07	(0.08)*	−0.03	(0.09)
				6 cm	−0.03	(0.06)*	0.08	(0.06)*	−0.06	(0.08)*
Δ*E*_CoM_	[BW *L*_0_]	0.02	(0.17)	4 cm	−0.05	(0.17)	0.03	(0.17)	−0.12	(0.20)*
				6 cm	−0.08	(0.14)*	0.01	(0.12)	−0.21	(0.28)*

Post-hoc t-test to compare the three step types −1, 0, and +1 to level running. Significant differences (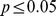) are indicated by asterisks.

As expected, some parameters of gait dynamics were significantly influenced by forward speed 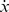 [32], [33] (table 1). For comparison to simulation results, we were interested in understanding the effect of the drop perturbation independent from variance in speed. For the factors that exhibited significant speed effect in the mixed model ANOVA, we further analyzed the speed effect using a simple regression analysis. We pooled the normalized data together (all birds, all trials and all step categories) and calculated each parameter's linear regression with respect to 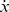, after confirming that the residuals from this regression were approximately normally distributed. If this analysis revealed a substantial speed effect by the criteria 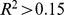 and 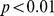, we recalculated the corresponding parameter (here, we use 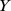 as a placeholder) by taking the residuals of the linear speed-regression (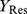) and adding the mean value of level running (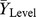):
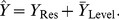
(1)Based on these results, for further analysis we used the speed-corrected leg length velocity 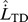 (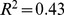, 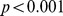) and the speed-corrected axial peak force 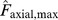 (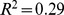, 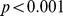).

### 3 Model

We used the passive, planar spring-loaded inverted pendulum (SLIP) as a reduced-order representation of whole-body dynamics of animal locomotion. This model is based on the observation that animals move with bouncing, spring-like gaits, with ground reaction forces approximated by a model with a point mass body and massless legs that resist only compressive loads [34]–[37]. This model has been widely used in biomechanics and robotics [38], because it qualitatively reproduces the dynamics of both walking [37] and running [34], [35]. The SLIP model is a passive, energy conservative dynamic template of locomotion [39]. While active stance models have also been suggested as templates for legged locomotion [40]–[45], the most appropriate choice of active stance model for running animals remains unclear. In this study, we are focused specifically on the influence of swing-leg trajectory on landing conditions and, consequently, the peak force and impulse of the leg during stance. The passive SLIP model provides good prediction of the stance peak force, impulse and overall body dynamics given specified landing conditions [5], [17], [34], [35], [37]. Consequently, the SLIP model is the most appropriate dynamic template for this study, because it allows us to focus specifically on the effects of swing-leg trajectory on running dynamics.

The SLIP model has a multitude of possible solutions, depending on initial conditions (body position and velocity) and leg parameters (leg stiffness and leg length). In this model, the body is represented by a point mass 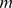 supported by a linear leg spring of stiffness 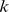 and resting leg length 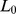, touching the ground with the angle of attack 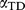 (figure 1(B)). During flight phase the CoM follows a ballistic curve, determined by the acceleration of gravity. The transition from flight to stance occurs when the landing condition 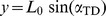 is fulfilled. During stance phase the equation of motion is given by
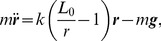
(2)with 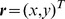 being the position of the point mass with respect to the foot point, 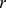 its absolute value and 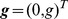 the gravitational acceleration, with 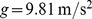. Take off occurs when the leg length (distance between the CoM and toe) exceeds the resting leg length 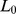. Since the system is energetically conservative, its state is fully described by the apex condition 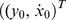, with 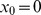 and 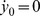 (the apex is the highest point of the CoM trajectory).

To estimate appropriate SLIP model leg stiffness 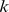 and TD angle of the virtual leg 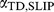, we optimized these model leg parameters to match the experimentally observed average ‘steady gait’ values for forward velocity, apex height, peak axial leg force and total axial leg impulse. As noted earlier, the peak force, impulse and body CoM dynamics of animal locomotion can be well approximated by the SLIP model [5], [17], [34], [35], [37]. In level terrain, all steady steps were included in the average used to fit a reference steady SLIP model. For the simulations in uneven terrain (4 cm and 6 cm drop), we used the step prior to the disturbance (step −1) to generate the reference steady gait, optimizing the leg parameters to match the peak force, axial impulse, apex height and forward velocity of this step. The model leg stiffness remained fixed within a terrain condition, and was therefore unchanged between step −1 and step 0, but was allowed to vary between terrains (level versus 4 cm, 6 cm drop runways), reflecting potential shifts in the reference ‘steady’ gait. The model was implemented in Matlab (R2012a, Mathworks Inc., Natick, MA, USA).

### 4 Running Simulations with Swing-Leg Trajectory

To simulate running, we used the SLIP model with initial conditions and parameters of the reference steady gait (see above), and applied a prescribed swing-leg trajectory as a function of fall time during the flight phase to control TD conditions at the swing-stance transition. We assume our model has an anticipated time of ground contact for the nominal steady gait at a given speed, but no specific information about the terrain, including the size and location of the drop. We prescribe a continuous evolution of swing-leg angle as a function of time during the flight phase, from the instant of apex until the actual ground contact (figure 1). This means that if the ground is contacted early or late compared to the reference steady gait, the TD conditions are altered.

During stance, no control was applied, and stance dynamics were solely determined by the TD conditions applied to the passive SLIP model. Thus, the only control applied to the model was the swing-leg trajectory as a function of fall time. We used the apex to initialize the swing-leg trajectory because it is a unique event that can be easily detected. Note, we do not assume any specific mechanisms of control to be analogous between the model and experiment. We are focused specifically on understanding how different swing-leg trajectories influence dynamics following a drop perturbation. A specified swing-leg trajectory could be achieved through a number of different control mechanisms, which are not the focus of the current study.

We generated optimized swing-leg trajectories based on three different objective functions for the subsequent SLIP-modeled stance phase: i) constant peak force, ii) constant axial impulse, or iii) equilibrium (steady) gait. For each proposed objective function, we solved for a swing-leg trajectory as a function of fall time based on the relationship between landing conditions and predicted stance phase dynamics using the SLIP model. We focused our attention specifically on the effects of swing-leg trajectory because previous experimental studies have suggested leg geometry at contact as a primary control target in running [5], [14], [16], [17], [46].

**Figure 2:**
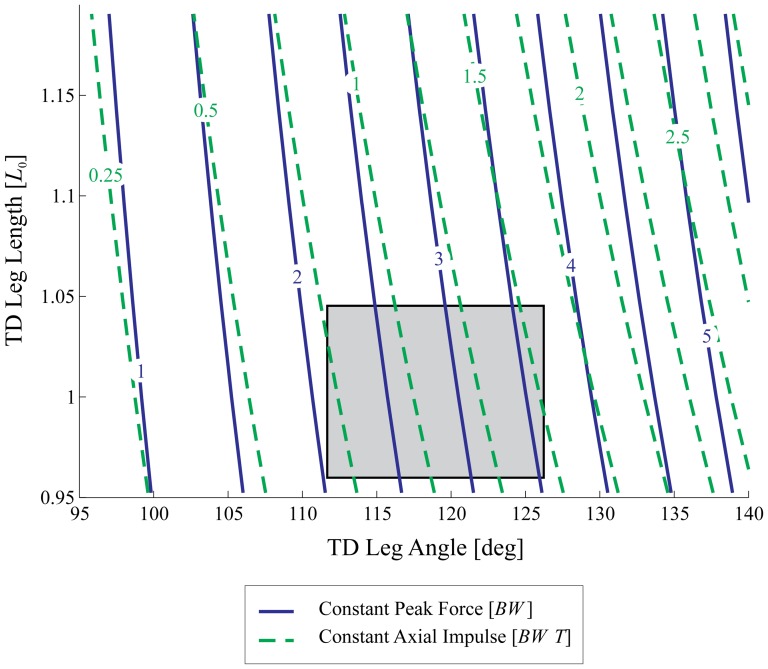
Swing-leg control strategy simulations of leg angle and leg length adjustment. Contours lines of constant peak force (blue solid lines) and constant axial impulse (green dashed lines) as a function of TD leg angle and TD leg length, predicted by the model simulations for one forward speed 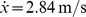 (experimentally observed average forward speed for level running). The gray square highlights the area of experimentally observed TD leg angles and TD leg lengths (lower and upper quartile). The slope of the contour lines reveals that TD leg angle has a much higher influence on both peak force and axial impulse than TD leg length. We subsequently focused our swing-leg control policies on leg angle adjustment only.

We performed an initial simulation analysis to reveal the consequences of simultaneous adjustment of swing-leg length and angle on the predicted stance peak force and axial impulse of the SLIP running model. Figure 2 shows the contour lines of constant peak force (solid lines) and axial impulse (dashed lines) as a function of TD leg angle and TD leg length, predicted by the SLIP model for a single forward speed 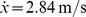 (average experimentally observed level running speed). Within the region of experimentally observed TD leg postures (gray square), the force and impulse contour lines are nearly vertically oriented (figure 2). This reveals that peak force and axial impulse are strongly influenced by leg angle at TD, whereas leg length at TD has a relatively small influence on SLIP-predicted stance leg loading. These observations suggest leg angle is the more effective target for swing-leg control of a SLIP running model. Furthermore, experimentally observed variation in leg length at touchdown is small in magnitude [17]. Animals tend to run with a consistent leg posture because variation in leg length influences gearing and muscle dynamics [10], [25]. Consequently, for simplicity, we focused our predictions on simulations of leg angle adjustment only, without changes in leg length.

Leg stiffness can also be adjusted as a function of fall time, as a potential control strategy for running [15]. However, leg stiffness is a stance parameter, and not a component of the ‘swing-leg trajectory’ *per se*. Although we did not directly investigate adjustment of leg stiffness as a function of fall time, we did nonetheless account for *between-terrain* shifts in leg stiffness, by fitting the nominal steady gait that observed in the ‘−1 step’ position on the runway (see Methods section 3). This allowed the nominal steady gait to change between terrains, but not from step-to-step. We did, however, measure the experimentally observed step-by-step variance in effective leg stiffness (see Results and Discussion).

In the ‘constant peak force policy’, we optimized the leg angle as a function of fall time such that the resulting peak force during stance remains constant for all steps. Specifically, we solve for a trajectory such that if the foot contacts the ground after apex, the landing conditions lead to a specified SLIP-modeled peak leg force. When this swing trajectory is applied to the model in the presence of a drop perturbation, the leg angle evolves until foot contact, and the peak force of the perturbed step (0) matches the peak force of the previous step (−1).

In the ‘constant axial impulse policy’, we regulate axial leg impulse rather than peak force, following similar methods. We solve for a swing-leg angular trajectory as a function of fall time to maintain a specific constant axial leg impulse achieved by the SLIP model. When this swing trajectory is applied to the model in the presence of a drop perturbation, the axial impulse of the perturbed step (0) matches that of the previous step (−1).

The ‘equilibrium gait policy’ has been suggested in theoretical literature as a method for achieving perfect disturbance rejection in uneven terrain [20], [30], [47]. This strategy ensures that the model achieves a steady gait (constant velocity and bounce height from apex to apex), with a symmetric CoM trajectories with respect to the vertical axis defined by mid-stance (TD and take off conditions are symmetrical). By choosing the appropriate TD leg angle for each velocity vector during the ballistic flight phase 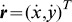, an equilibrium gait is obtained regardless of when the foot contacts the ground. We used this relationship to solve for a leg angle trajectory as a function of fall time to ensure steady gait of the SLIP model. While birds may not use a perfect equilibrium gait running, we consider the possible strategy that they optimize swing-leg trajectory to minimize deviations from an equilibrium gait for disturbance rejection.

Further analysis of simulations using the methods above, as well as discussion of stability implications, can be found in Vejdani et al. 2013 [20].

## Results

We first report the experimentally observed changes in running dynamics (section 1), followed by a description of the simulation predictions (section 2), and comparison between experimental results and simulation predictions (section 3).

### 1 Experimental Data

**Figure 3:**
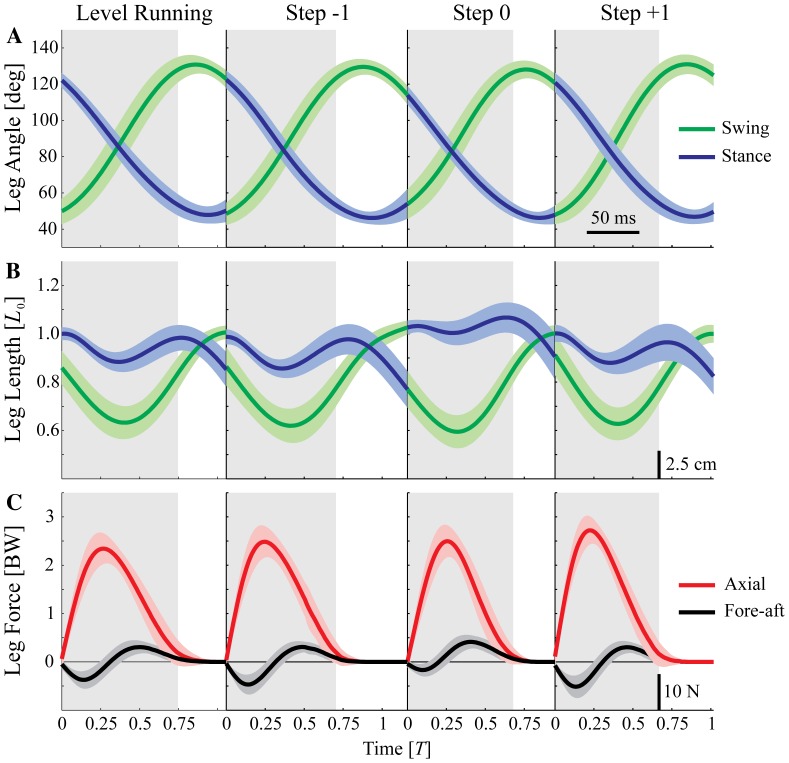
Experimental data: trajectories over time. Mean values (solid lines) and standard deviation (colored area) of leg angle (A), and leg length (B) (stance leg in blue, swing leg in green), and leg force (C) (axial force in red, fore-aft force in black) against step time for level running and the three step types −1, 0, and +1. The gray areas indicate the stance phases. The leg angle of both stance and swing leg follows a sinusoidal trajectory (A). Compared to the other step types, the compression of the stance leg is lower during the drop step (step 0) (B). In the drop step (step 0), the axial peak force is not significantly different from the previous step (step −1) or level running, but the fore-aft force indicates an acceleration (C).

**Figure 4:**
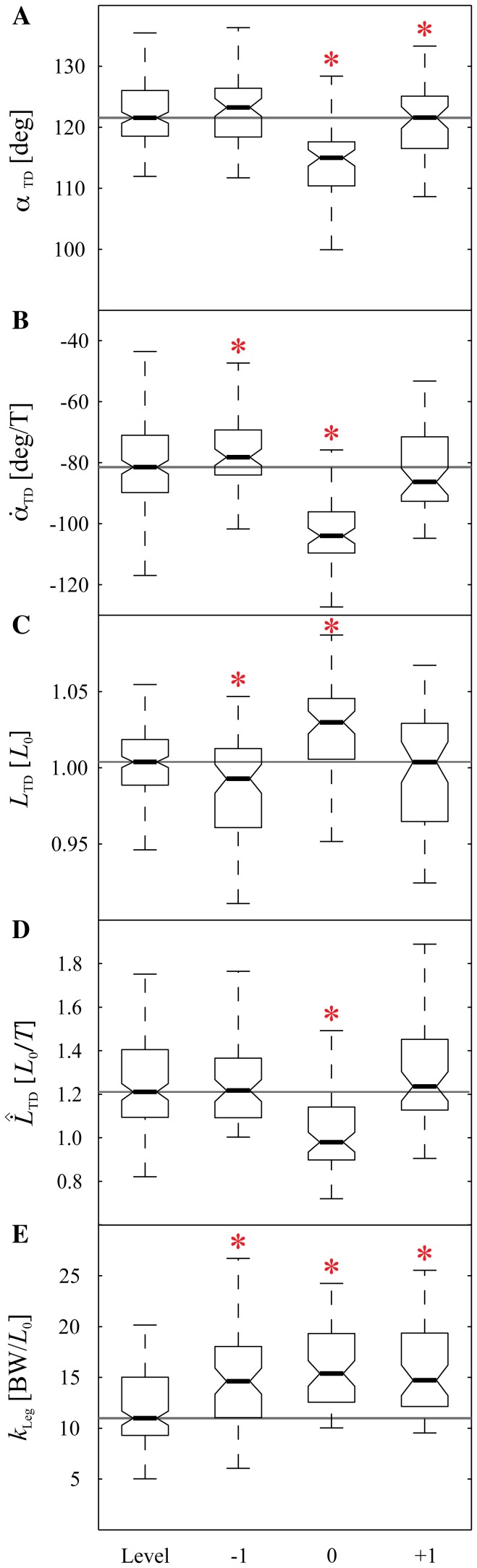
Experimental data: landing conditions. Boxplots of five TD parameters leg angle 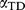 (A), leg angular velocity 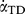 (B), leg length 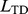 (C), speed-corrected leg length velocity 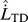 (D), and leg stiffness 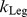 (E) for level running and the three step types −1, 0, and +1. The boxes indicate the median (black line) and the range between the lower quartile (Q1) and the upper quartile (Q3). The whiskers show the range between the lowest and the highest value still within 1.5× IQR (inter quartile range IQR = Q3 - Q1). For simplicity, individuals and drop heights have been pooled together (see table 2 for more detailed information). Asterisks indicate a significant difference (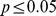) compared to level running (post-hoc t-test). The drop step (step 0) differs significantly from level running for all five variables.

**Figure 5:**
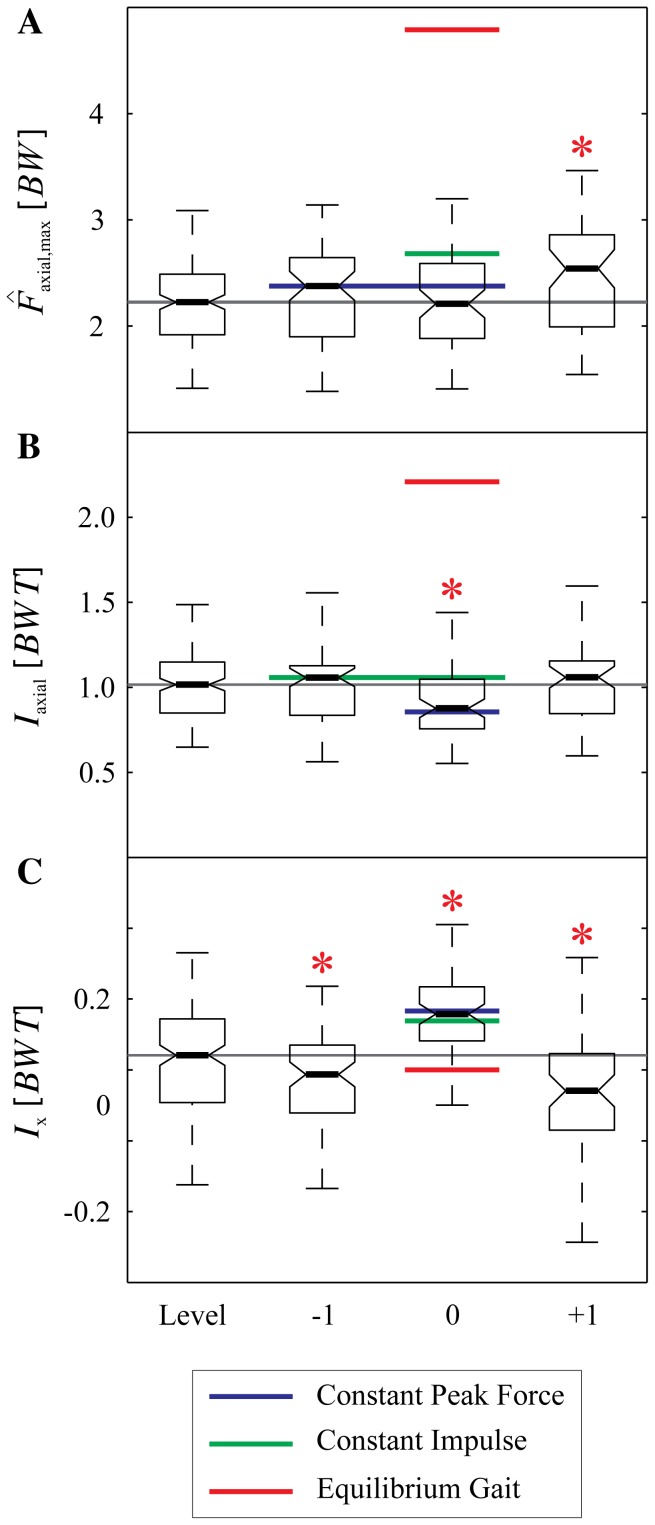
Experimental measures of stance dynamics, overlaid with simulation predictions. Boxplots of three stance measures from the running birds: speed corrected axial peak force 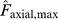 (A), axial impulse 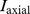 (B), and fore-aft impulse 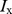 for level running and the three step types −1, 0, and +1. Asterisks indicate a significant difference (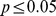) compared to level running. See tables 1 and 2 for more detailed statistical results. The colored lines show the simulation predictions for the three swing-leg control policies applied to the drop step: constant peak force (blue), constant impulse (green), and equilibrium gait (red). Swing-leg trajectories optimized for equilibrium gait predict higher 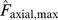 (A) and 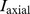 (B) during the drop step (step 0), which is not experimentally observed. Swing-leg trajectories optimized for constant peak force or constant impulse both result in a good match between measured and predicted dynamics. Analysis of simulation fits across all drop perturbation trials suggest these two policies are equally good at predicting changes in landing conditions (table 3, figure 7).

When guinea fowl negotiate an anticipated drop step, the trajectories over time of leg angle and axial leg force remain remarkably similar to level terrain locomotion. Figure 3 shows the measured trajectories over time of the birds' leg angle (A), leg length (B), and leg force (C) for the different step types (Level, −1, 0, +1). Notable shifts occur in the stance fore-aft impulse and leg length trajectory of step 0 (figure 3). The results of the ANOVA for experimentally measured variables are listed in table 1, with post-hoc pairwise comparisons in table 2 and boxplots of data in figures 4 and 5. These findings are summarized below.

#### Swing-Leg Kinematics in the Drop Step

The leg angle follows a consistent sinusoidal trajectory (figure 3(A), blue: stance leg, green: swing-leg), with little apparent change during negotiation of the drop step. Nonetheless, landing conditions vary in the drop step due to the extension of the ballistic flight phase at the transition between steps −1 and step 0. In the elongated flight phase, continuing leg retraction causes the bird to land with a steeper leg angle 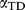 at step 0 compared to level running (table 1). The leg length trajectory (figure 3(B), green line) also shows a relatively consistent trajectory across the the step types, but with a slowed rate of lengthening during the elongated flight phase. This results in a small but significant increase in leg length 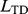, but a decrease in leg velocity 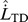 at touchdown in step 0 compared to level running.

#### Anticipatory Changes in Step −1

In step −1, preceding the drop, the leg length 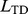, leg angular velocity 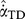 and leg length velocity 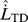 differ slightly but significantly from level terrain running (figure 4 and table 2). These findings suggest the birds tune their gait in anticipation of the drop step, which has also been observed during negotiation of visible obstacles [13]. In step −1, the birds reduced leg retraction speed, adopted a 1–2% more crouched leg posture, and increased effective leg stiffness. The increase in leg stiffness was maintained across all three steps of the drop terrain (steps −1,0,+1), whereas the other leg parameters varied between steps (table 2) across the drop terrain. Nonetheless, the swing-leg trajectories remain very similar to level running (figure 3), suggesting that the overall task-level swing-leg control strategy may be maintained across step types within each terrain, with variation in the timing of ground contact causing step-by-step variations in landing conditions.

#### Body Dynamics and Stance Leg Forces

The body dynamics during negotiation of the drop (step 0) are very similar to those observed by guinea fowl negotiating an unexpected pothole [17]. The axial peak force remains consistent in the step preceding (step −1) and during the perturbation (step 0), with no statistically significant change until step +1 (figure 3(C), and table 2). The total axial impulse 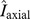 (integral of force over time) does not change in step −1, but decreases slightly in step 0, due to reduced stance duration. The net fore-aft impulse indicates acceleration in step 0 (figure 5 and table 2), but 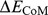 does not differ significantly from level terrain. This indicates that gravitational potential energy of the drop is passively converted to forward kinetic energy, increasing velocity, similar to unexpected pothole experiments [17]. The increased velocity is not maintained, because the negative fore-aft impulse 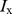 and the negative net CoM work 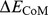 in the subsequent step (step +1) indicate that the bird actively absorbs energy, slowing down (table 2).

In the step preceding the drop (step −1), the net fore-aft impulse 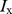 indicates slight deceleration, and the net change in body CoM energy 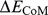 is slightly negative (figure 5 and table 2). Thus, the results indicate a small active deceleration in anticipation of the drop.

### 2 Simulation Results

We generated optimized swing-leg trajectories based upon three hypothesized task-level priorities: i) constant peak force, (ii) constant impulse, and iii) equilibrium (steady) gait. The optimized swing-leg trajectories were applied to a simple running model (see Methods section 3) to predict the swing and stance dynamics in ‘step 0’ of the drop perturbation.

**Figure 6:**
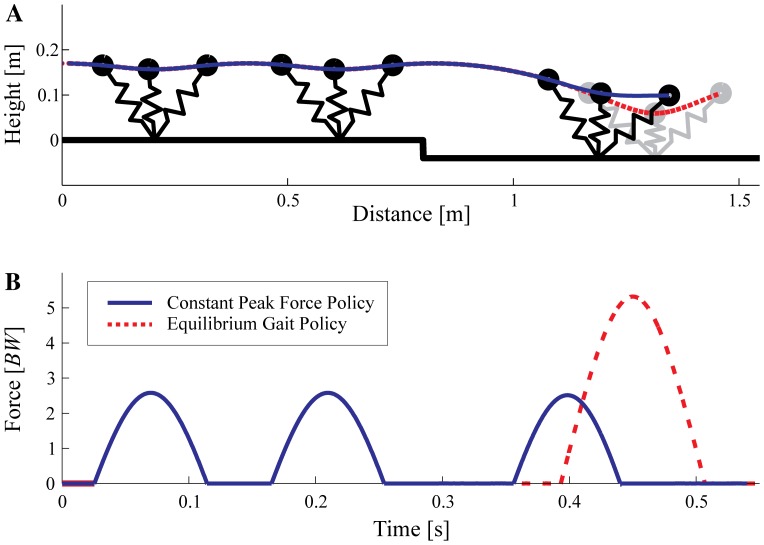
Representative simulations illustrating the divergence between equilibrium gait (steady gait) and constant peak force control strategies. CoM trajectories (A) and force profiles (B) of the simulation results for two swing-leg control strategies: constant peak force (blue solid lines) and equilibrium gait (red dashed lines). The equilibrium gait strategy achieves steady dynamics but demands high forces; whereas the constant peak force strategy results in non-steady dynamics in the drop step, and requires adjustment in subsequent steps to return to a steady gait.

The simulations of swing-leg trajectory targeting constant peak force and constant impulse predict relatively similar dynamics during the drop step (table 3 and figure 5). As an illustration of the simulation results for a drop perturbation, figure 6 shows the CoM trajectories (A) and force profiles (B) of the SLIP model with two swing-leg control strategies—constant peak force (solid lines), and equilibrium gait (dashed lines). During level running the CoM trajectories and force profiles are identical, but when the flight phase duration differs from the expected nominal steady gait, the predictions of the two control strategies diverge. The predicted peak force and axial impulse in the drop step increase drastically for the equilibrium gait strategy. This lends further evidence to the trade-off suggested from previous theoretical studies (see Introduction). The constant peak force and constant impulse control strategies both result in a non-steady stance in the drop step, indicated by a positive fore-aft impulse (figure 5, green and blue lines). Thus, gravitational potential energy from the drop perturbation is converted into horizontal kinetic energy, and the running model accelerates. This forward acceleration is in agreement with the experimentally observed dynamics (figure 5C).

**Table 3:** Simulated control strategies compared to experimental data.

	Control Policy	Δ*α*_TD_ [deg]	RMSE [deg]	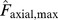 [BW]	*I*_axial_ [BW *T*]	*I*_x_ [BW *T*]
Level	Constant Peak Force	−0.6	5.4	2.33	1.00	0.03
	Constant Impulse	−0.5	3.1	2.46	1.08	0.02
	Equilibrium Gait	2.0	5.9	2.56	1.20	0
Step −1	Constant Peak Force	−0.2	5.0	2.42	1.03	0.04
	Constant Impulse	−0.4	3.0	2.53	1.10	0.03
	Equilibrium Gait	2.5	5.6	2.80	1.22	0
Step 0	Constant Peak Force	−0.3	4.5	2.42	0.92	0.08
	Constant Impulse	2.0	4.2	2.75	1.10	0.06
	Equilibrium Gait	9.0	10.7	4.79	2.21	0

Difference 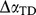 and root mean squared error (RMSE) of the predicted virtual leg angle at TD 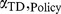 and the experimentally measured virtual leg angle at TD 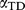. Axial peak force 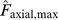, axial impulse 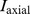, and fore-aft impulse 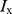 are the predicted values of the corresponding control strategies. Compared to the equilibrium gait strategy, the RMSE suggest that both constant peak force and constant impulse control predict the TD leg angle more accurately.

### 3 Comparison between Experimental Data and Simulation Results

**Figure 7:**
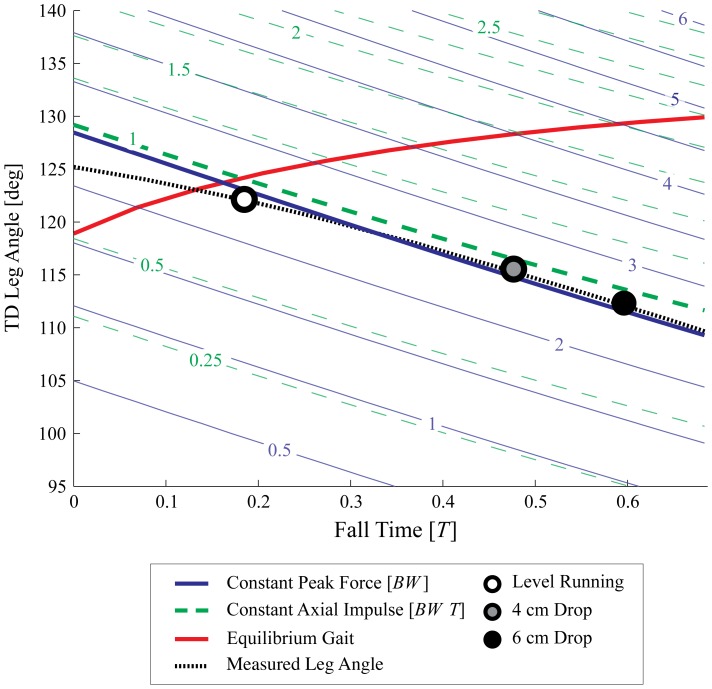
Model predicted late-swing leg angular trajectories, in comparison with experimental data. Predicted swing-leg trajectories, shown as leg angle against fall time (time from apex until TD), derived from SLIP simulations to achieve constant peak force (blue solid lines), constant axial impulse (green dashed lines), or equilibrium gait (red line) at touchdown. Predictions are for a single forward speed 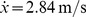. The thick peak force (blue) and impulse (green dashed) contours indicate the predicted swing-leg trajectories, with thinner contours illustrating the gradient in force and impulse as the trajectory deviates from this. The mean measured leg trajectory is overlaid (dotted black line), along with the mean TD conditions for level running (white circle), 4 cm drop (gray circle) and 6 cm drop (black circle). The equilibrium gait trajectory (red) crosses loading contours, leading to increased force and impulse. The linearity of the constant peak force and impulse contours indicates that these strategies can be approximated by leg retraction with a constant angular velocity, whereas equilibrium gait requires leg protraction. The experimental data follows constant loading contours, suggest that guinea fowl do not use swing-leg trajectory to target equilibrium gait.

Simulations of constant peak force or constant impulse policies both result in a reasonably good match between measured and predicted dynamics. The constant peak force policy provides a slightly better match to median peak forces and axial impulse; however analysis of simulation fits across all drop perturbation trials suggest these two policies are equally good at predicting changes in landing conditions (table 3, figure 7). Consequently, we cannot conclusively distinguish between them.

To quantitatively compare simulation predictions to experimental data, the most relevant parameters are TD leg angle in step 0 and the predicted changes in stance dynamics resulting from the altered landing conditions. The TD leg angle is predicted by applying the optimized swing-leg angular trajectory during the ballistic flight phase. The simulations allow us to evaluate the interaction between swing and stance dynamics, and identify aspects of bird running that match and deviate from the model predictions. To determine which swing-leg control policy was most consistent with guinea fowl behavior, we ran a simulation for each running trial, predicting the drop step dynamics by applying the three control policies to the SLIP model as described in the methods (Methods section 4). For each control policy, table 3 reports the average differences 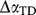 and root mean squared errors (RMSE) between the predicted touchdown virtual leg angle 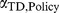 and experimentally measured 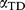 (Methods section 3). Compared to equilibrium gait, both constant peak force and constant axial impulse control result in smaller deviations between predicted and measured TD leg angle 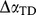 and smaller RMSE, suggesting a more accurate prediction of the TD leg angle across all three step types simulated (level, −1 and 0).

Stance phase peak force 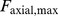, axial impulse 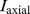, and fore-aft impulse 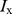 were simulated by applying the TD conditions resulting from each swing-leg control policy to the SLIP model (table 3). The simulation predictions are compared to experimental data in figure 5, with boxplots showing the distribution of experimental data and colored lines indicating predictions of each control strategy. Simulations of the equilibrium gait policy predict considerable increases in 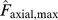 and 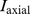 during the drop step (step 0), which is not experimentally observed (figure 5).

To further illustrate the divergence between the force and equilibrium gait policies, figure 7 shows the swing-leg trajectories predicted by the different control strategies for one constant forward speed 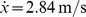 (average experimentally observed forward speed). Contour lines of constant peak force (blue lines) and constant axial impulse (green lines) are plotted as a function of TD leg angle (y-axis) and fall time (x-axis), indicating the trajectories for each control policy (a single predicted swing-leg trajectory follows a single contour line). The red line indicates the leg angle trajectory that leads to equilibrium gait (here indicating swing-leg protraction as a function of fall time). The experimentally measured TD leg angles are shown for level running (white circle), 4 cm drop (gray circle) and 6 cm drop (black circle). The experimentally observed TD conditions lie between contour lines for constant peak force (blue) and constant axial impulse (green), but differ markedly from the predictions of equilibrium gait. The approximate linearity of the contour lines for constant peak force and constant axial impulse indicate that these policies can be closely approximated by retracting the leg with a constant angular velocity (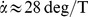 for peak force control, and 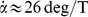 for impulse control at the representative forward velocity shown).

**Figure 8:**
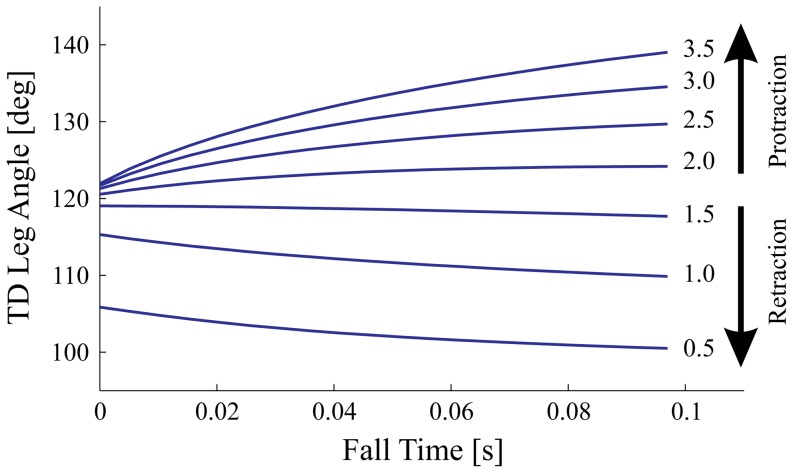
Late-swing leg angular trajectories predicted for the equilibrium gait policy, targeting steady gait. The equilibrium gait policy predicts a shift from late-swing retraction to protraction with increasing speed. Shown are the swing-leg angle trajectories predicted from simulations optimized for equilibrium gait, for a range of speeds 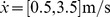. The simulations predict late-swing leg retraction for low speeds (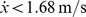), and protraction for higher speeds (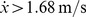).

For the equilibrium gait policy, the simulation predicted swing-leg angular trajectory varies between late-swing retraction and protraction, depending on forward speed. Figure 8 shows the simulation predicted swing-leg angle trajectories resulting in equilibrium gait for forward speeds between 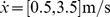, with constant leg length and leg stiffness. The simulations predict late-swing retraction for low speeds (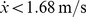), and protraction for higher speeds(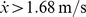). For a system with the body mass and virtual leg length of a guinea fowl, running at a forward speed of 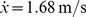, an equilibrium gait can be achieved with a constant leg angle (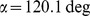), without adjusting the leg angle during swing (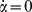). Within the observed speed range of guinea fowl, the equilibrium gait policy predicts late-swing protraction. Yet, experimental data show that birds consistently retract their legs in late swing (table 1 and figure 4) across all speeds and step types.

Although experimentally observed TD leg angles for steady level running (white circle) lie close to equilibrium gait predictions (red line), there is no evidence that the swing-leg trajectory directly targets equilibrium gait, because the drop perturbations lead to a sharp deviation from equilibrium gait predictions. Instead, the results suggest that the guinea fowl behavior more closely match predictions of swing-leg trajectory optimized to maintain constant peak leg force or constant leg impulse.

## Discussion

Perturbation experiments [13], [17] and theoretical models of walking and running [14]–[16], [18]–[20], [22] have suggested swing-leg trajectory as a critical target of control for legged locomotion because stance dynamics are highly sensitive to landing conditions. Swing-leg trajectory influences the timing of ground contact, the landing leg posture and body velocity at contact. These landing conditions, in turn, influence stability [14]–[16], [18], robustness [19], leg work [19], [20], disturbance rejection and collision impact energy losses [18]. Swing-leg trajectory can be optimised for consistent leg loading and economy, or alternatively, for steady body dynamics, but not all of these simultaneously [15], [16], [18]–[20]. We investigated how running guinea fowl manage this potential trade-off by measuring their ‘optimized’ locomotor strategy for negotiating a visible and well-practiced step down in terrain. The simulation results in figures 6 and 5 provide further evidence of the suggested trade-off in swing-leg trajectory. The specific swing-leg angular trajectory used by running guinea fowl is consistent with task-level priority to regulate leg loading (limiting fluctuations in peak force and impulse), rather than priority to maintain steady body dynamics. The birds' swing-leg angular trajectory is consistent with both the constant peak force and constant impulse policies, but clearly deviates from the predictions of the equilibrium gait policy.

The constant peak force and constant leg axial impulse policies both predict leg retraction in late swing with nearly constant angular velocity (figure 7). Previous studies have shown that running animals tend to retract the leg in late swing [14], [17], [48]; however, these studies could not explain the specific leg retraction velocities used by animals, because a wide range of retraction velocities can provide stability [14]–[16]. ‘Stability’ simply refers to whether or not the system recovers—whether a deviation in body dynamics decays (stable) or grows (unstable) over time [49], [50]. Priority for stability alone is not sufficient to predict a specific leg angular trajectory. The equilibrium gait policy predicts a specific leg angular trajectory by targeting a perfectly steady gait, which can theoretically provide perfect disturbance rejection in the face of terrain height variation [20], [47]. However, this policy can demand large increases in force and impulse in the stance phase. Furthermore, the equilibrium gait policy can predict either leg protraction or retraction of the leg in late swing (figure 8). While stable spring mass running with swing-leg protraction is possible (with appropriately tuned leg stiffness) [16], this strategy would result in higher leg impacts due to increased velocity of the foot with respect to the ground [15] (e.g., the opposite of ‘ground speed matching’, [48]). This might explain why, to our knowledge, only swing-leg retraction, never protraction, has been experimentally observed in bipedal locomotion of humans [51] and birds [12], [16], [17].

We found that stance dynamics immediately following the drop perturbation (step 0) are consistent with a passive energy-conservative leg model, albeit with a non-steady response in which gravitational potential energy is converted to kinetic energy, causing forward acceleration. In fact, the overall body dynamics of step 0 are remarkably similar to those of an unexpected drop step [17], despite evidence of anticipatory changes to gait in the drop terrain. The anticipatory adjustments include small but significant changes in the nominal gait of step −1 preceding the drop (table 2), and an increase in effective leg stiffness across all steps in the drop terrain (figure 4). These findings suggest that guinea fowl tune gait dynamics depending on context including the anticipated ‘roughness’ of terrain.

Nonetheless, leg angular trajectory remains remarkably constant and rhythmic across steps within each terrain (figure 3), suggesting that birds target a consistent optimized trajectory within a terrain context and avoid step-by-step adjustments. A prescribed swing-leg trajectory has potential to be implemented through feed-forward control, with minimal feedback, circumventing neuromuscular delays. However, our results do not reveal the underlying neural control mechanisms used to achieve the observed swing-leg trajectory. A consistent leg angular trajectory could be achieved through a combination of feedforward and feedback mechanisms, making use of internal models of dynamics as well as vestibular, visual and proprioceptive sensory information. Whatever the underlying control mechanisms, our findings are consistent with the idea that animals optimize swing-leg trajectory to achieve well-defined intrinsic-dynamic characteristics at the swing-stance transition, to bridge neuromuscular delays and minimize the need for rapid neural modulation.

Although the results confirm that step 0 dynamics can be well approximated by a passive, energetically conservative leg model, the dynamics of the 2nd stance (step +1) clearly indicate net energy absorption, which cannot be achieved with a passive model. Consequently, a full dynamic model of the birds' recovery over several steps requires a more sophisticated stance leg model that includes actuation. It will be interesting in future work to further investigate alternative task-level templates of running that allow for non-conservative stance dynamics following terrain perturbations. Actuated template models have been proposed and analyzed from a theoretical perspective [40]–[45], but it is not yet clear which of these is most appropriate for animal legged locomotion. Elaborations of stance models were not considered here because we were primarily focused on the effects of swing-leg trajectory on the swing-stance transition. Non-conservative stance models would have confounded the interpretation of swing-leg trajectories. The initial step down response (step 0) is energetically conservative and matches well with SLIP leg loading predictions, so we concluded that a more complex model was not justified for the current study. Nonetheless, future work should investigate more complex stance models to further explore the interactions between swing and stance dynamics in non-steady locomotion, in particular to understand the full time course of recovery from a perturbation.

Additionally, we observed asymmetry in the force trajectory across all running conditions—which has also been noted previously [52] and likely reflects the complex underlying musculoskeletal structure and dynamics of animal legs. The passive SLIP model does not predict the precise shape of the biologically observed leg force trajectory, because it is also influenced by factors such as damping in tissues, muscle contractile properties and musculoskeletal gearing effects. The SLIP model serves only as a general ‘template’ of the overall body dynamics of running gaits [39], and does not reflect the specific underlying neuromuscular and musculoskeletal mechanisms. Nonetheless, template models such as SLIP provide a convenient approximation of legged locomotion because animals tend to use periodic gaits with ground reaction forces and body dynamics that can be approximated by a point mass body with massless legs that resist only compressive loads [34]–[37]. The SLIP model is not the only model that provides a reductionist approximation of locomotor dynamics [40]–[45], [49], [53]–[56]; however, it is the most widely validated choice for simulations of running (see Methods section 3). These caveats aside, we have found that a simple reductionist model can reproduce many aspects of avian running dynamics during negotiation of a drop in terrain, by optimizing swing-leg angular trajectory to target landing conditions that meet the specific task-level priority of regulating stance leg loading.

The observed strategy of minimizing fluctuations in peak force and impulse may also minimize energy cost of transport. Cost of transport is influenced by both muscular force and work [6], [56], which is therefore strongly related to ground reaction force [57]. Additionally, a separate simulation study has compared swing-leg trajectories optimized for force, impulse and leg work, and found that all three of these policies predict similar swing-leg trajectories, yet diverge from the predictions of an equilibrium gait policy [20]. Thus, it appears that load regulation and economy are closely aligned priorities. A swing-leg trajectory optimized to regulate leg loading may have the dual benefits of minimizing injury risk and maximizing economy of uneven terrain locomotion.

The majority of animal locomotion studies have focused on steady-state locomotion, and many studies either implicitly or explicitly assumed that steady gait is an overriding priority and therefore a direct target of active control. While animals must avoid falling in uneven terrain, ‘stability’ and ‘disturbance rejection’ or ‘steadiness’ of gait may not be exceptionally pressing priorities for the control of swing-leg trajectory compared to other task-level demands, such as injury avoidance and economy. Applying a swing-leg trajectory that enforces a steady gait could dramatically increase the peak force and impulse experienced by the leg in the presence of a terrain drop. These forces could easily exceed the safety factors of animal musculoskeletal tissues, which are around 2–4× peak force of steady locomotion [25], [26]. Therefore, minimizing fluctuations in peak force and impulse to prevent damage to musculoskeletal structures might be a more pressing priority than immediate recovery to a nominal steady gait following perturbations.

Nonetheless, disturbance rejection is likely an important priority over slightly longer timescales. This conclusion is supported by the experimental finding that guinea fowl consistently recover from terrain perturbations within about 2–3 strides [10], [11], [13], but do not exhibit perfect, immediate disturbance rejection, even for small terrain perturbations [13]. We suggest that the immediate imperatives of swing-leg control in animal legged locomotion are related to injury avoidance and economy, not immediate stabilization to a nominal steady gait, while stance phase mechanisms (e.g., energy absorption/insertion) facilitate recovery to steady gait over multiple steps.

Our simulations suggest a simple method for generating target swing-leg trajectories for implementation in legged robots to achieve performances similar to that of running animals. The ‘equilibrium gait’ policy has been suggested for legged robots for its disturbance rejection properties [30], [47]; however, we suggest that it may be undesirable for systems with significant force limitations. A separate recent paper explores in more detail simulations of running dynamics with multiple alternative swing-leg control policies [20], and this paper also further discusses potential implications for bio-inspired robots. This systematic approach of comparing predictions based on multiple potential task-level priorities could help engineers design and control robots to benefit from passive-dynamic structures, minimize actuator demands and minimize control effort.

## Conclusions

We have presented a novel approach combining simulations and experiment that allows us to investigate the task-level priorities in non-steady animal locomotion, including disturbance rejection, injury avoidance and economy. Guinea fowl negotiate a downward step using unsteady dynamics with forward acceleration, and recover to steady gait in subsequent steps. ‘Steadiness’ of gait does not appear to be the direct or immediate priority governing swing-leg trajectory used by running animals. Our results suggest, instead, that guinea fowl use swing-leg trajectories that reflect priority for load regulation, which may facilitate injury avoidance and economy in uneven terrain.

## Supporting Information

Text S1 **List of symbols, terms and definitions.**
(PDF)
