# Supplementary material for: Swing-Leg Trajectory of Running Guinea Fowl Suggests Task-Level Priority of Force Regulation Rather than Disturbance Rejection
Source: PLoS One. 2014 Jun 30;9(6):e100399. doi: 10.1371/journal.pone.0100399 (PMC4076256; doi:10.1371/journal.pone.0100399)
Supplement: Text S1 — List of symbols, terms and definitions. (PDF) [file pone.0100399.s001.pdf]

List of abbreviations, definitions and symbols

Abbreviations

CoM	center of mass
SLIP	spring loaded inverted pendulum model
TD	touch down, referring to landing conditions at the swing-stance transition

Definitions

Model	A reduced-order mathematical description of the physical system. Here we use the highly reductionist spring-mass model with massless leg.
Landing conditions	Initial conditions of the CoM (position and velocity) at the beginning of stance. These are directly influenced by the swing-leg trajectory during flight.
Passive dynamics	Synonymous with intrinsic dynamics—the response of the physical model. Here, the stance dynamics of the model are fully determined by landing conditions and leg stiffness.
Control policy	Active control applied to the model with a specific target performance goal. Here the only applied control is late-swing leg angular trajectory.
Peak force control	Late-swing leg trajectory optimized to target landing conditions for constant peak force of the SLIP model in the drop step (equal to the peak force of the previous step).
Impulse control	Late-swing leg trajectory optimized to target landing conditions for constant axial impulse of the SLIP model in the drop step (equal to the impulse of the previous step).
Equilibrium gait control	Late-swing leg trajectory optimized to target landing conditions for perfect disturbance rejection of the SLIP model in the drop step, resulting in a steady, symmetric gait cycle.

Parameters

SI Units

g	gravitational acceleration [m/s^2]
m	body mass [kg]
L_0	resting leg length [m]
$\mathrm{BW} = mg$	body weight [N]
$T = \sqrt{L_0/g}$	periodic time of a pendulum [s]

Non-dimensional

α	leg angle [deg]
α_{Policy}	angle of the virtual leg (CoM to foot) predicted by a swing-leg control strategy [deg]
α_{SLIP}	angle of the virtual leg for the SLIP model during stance [deg]
$\dot{\alpha}$	leg angular velocity [deg/T]
ΔE_{CoM}	net CoM work [BW L_0]
F_{axial}	axial leg force [BW]
I_{axial}	axial leg impulse [BW T]
I_{x}	fore-aft impulse [BW T]
k_{Leg}	effective linear leg stiffness [BW/L_0]
L	leg length [L_0]
$\dot{L}$	leg length velocity [L_0/T]
$\boldsymbol{r} = (x, y)^T$	CoM position [L_0]
$\dot{\boldsymbol{r}} = (\dot{x}, \dot{y})^T$	CoM velocity [L_0/T]
$\ddot{\boldsymbol{r}} = (\ddot{x}, \ddot{y})^T$	CoM acceleration [L_0/T^2]
t	time [T]
